# Tumor regression grades in locally advanced gastroesophageal adenocarcinoma: associations with survival, pathological risk factors, and molecular biomarkers in a 19-year real-world cohort

**DOI:** 10.1016/j.esmogo.2026.100408

**Published:** 2026-09-02

**Authors:** H.C. Puhr, B. Mozayani, M. Korpan, M. Kaya, F. Kordik, M. Kastner, A. Duman, G. Jomrich, D. Kollmann, S.F. Schoppmann, J.M. Berger, G.W. Prager, E.S. Bergen, M. Preusser, A. Ilhan-Mutlu

**Affiliations:** 1 Department of Medicine I, Division of Oncology, Medical University of Vienna, Vienna, Austria; 2 Department of Pathology, Medical University of Vienna, Vienna, Austria; 3 Department of General Surgery, Division of Visceral Surgery, Medical University of Vienna, Vienna, Austria

**Keywords:** esophageal cancer, gastric cancer, gastroesophageal junction cancer, survival, tumor regression

## Abstract

**Background:**

Tumor regression grade (TRG) after neoadjuvant therapy has been proposed as a prognostic marker in resectable gastroesophageal adenocarcinoma, yet its independent value beyond pathological staging remains uncertain.

**Materials and methods:**

This retrospective single-center study included 692 patients with locally advanced, resectable gastroesophageal adenocarcinoma treated between 2006 and 2024 at the Medical University of Vienna. Among 394 patients receiving neoadjuvant systemic therapy, 175 had documented TRG according to Mandard and/or Becker. Temporal trends in treatment and TRG reporting were assessed. Associations between TRG, clinicopathological factors, mismatch repair, programmed cell death receptor ligand 1, and human epidermal growth factor receptor 2 status were analyzed. Disease-free survival (DFS) and overall survival (OS) were evaluated using Kaplan–Meier and univariable Cox regression models.

**Results:**

The use of neoadjuvant/perioperative chemotherapy and documentation of TRG increased substantially over time. Tumor regression was heterogeneous, with a predominance of poor responders. Lower TRG scores were strongly associated with favorable ypT and ypN categories and absence of lymphatic, venous, and perineural invasion (all *P* < 0.05). Mandard TRG was significantly associated with DFS (*P* = 0.03) and OS (*P* = 0.0015), whereas Becker TRG was not. Classical pathological factors (ypT, ypN, ypV, ypPn, and R) remained the strongest prognostic determinants. The neoadjuvant treatment strategy was associated with TRG scores. Multivariable analysis was limited by event numbers and missing data.

**Conclusion:**

In this long-term real-world cohort, Mandard TRG was associated with survival, although its prognostic impact appears closely linked to pathological stage and molecular context. Prospective studies integrating standardized TRG assessment with pathological TNM (tumor–node–metastasis) staging and biomarkers are warranted, particularly in the evolving era of perioperative chemo-immunotherapy.

## Highlights

• Mandard TRG was associated with DFS and OS, whereas Becker TRG was not.
• Lower TRG scores were associated with favorable ypT/ypN and less invasive features.
• Classical pathological factors remained the strongest prognostic determinants.
• Neoadjuvant treatment strategy was associated with TRG.
• Most patients showed poor pathological response despite neoadjuvant therapy.

## Introduction

Gastroesophageal adenocarcinoma is a leading cause of cancer-related mortality worldwide,^1^ with a substantial number of patients presenting with locally advanced disease that carries a high risk of recurrence and cancer-related death despite multimodal treatment. Over the past two decades, perioperative chemotherapy has become the standard of care for resectable gastroesophageal adenocarcinoma after pivotal trials demonstrated a survival benefit compared with surgery alone.^2^

Pathologically documented response (pathological response) to neoadjuvant treatment in resection specimens, commonly quantified as tumor regression grade (TRG), has emerged as an important surrogate marker of treatment efficacy and a potential prognostic factor in upper gastrointestinal malignancies.^3^^,^^4^ Several systems are used in clinical practice to distinguish good and poor responders, with the Mandard and the Becker TRGs as the most commonly used in Western countries.^5^ The Mandard TRG, which was initially developed for esophageal cancer, focuses on the balance between fibrosis and residual tumor cells,^6^ whereas the Becker TRG, originally proposed for gastric cancer, estimates the percentage of residual viable tumor.^7^

Many studies report that major regression (low TRG) associates with lower ypT and ypN stage. It also tends to correlate with less lymphovascular and perineural invasion and better survival. Thus, pathological response rate, especially complete pathological response (pCR), is a common primary endpoint in phase II trials and a secondary endpoint in phase III trials.^8^ However, results concerning the prognostic value of TRG scores are not entirely consistent,^3^^,^^9^^,^^10^ and the independent prognostic value beyond ypTNM stage remains uncertain. Although some studies have investigated the potential adaptation of adjuvant therapy based on TRG, TRG currently has no direct consequences for adjuvant treatment decisions.^2^^,^^11^^,^^12^

As biomarkers such as human epidermal growth factor receptor 2 (HER2) expression, mismatch repair (MMR) status, and programmed cell death receptor ligand 1 (PD-L1) expression influence tumor biology, immune microenvironment, and clinical outcome, they are gaining importance also in localized settings.^13^, ^14^, ^15^ More recently, the addition of durvalumab to perioperative fluorouracil, leucovorin, oxaliplatin, docetaxel (FLOT) chemotherapy has emerged as a new standard of care in fit patients, reflecting the growing role of immunotherapy.^16^ However, the prevalence of these biomarkers as well as the addition of targeted therapy and checkpoint inhibitors in association with histopathological regression and clinical outcome in locally advanced gastroesophageal adenocarcinoma has not yet been investigated thoroughly in real-world cohorts.^10^

Thus, there is a need for large, real-world cohorts with long follow-up to clarify how TRGs, classical pathological factors, and biomarkers such as HER2, MMR, and PD-L1 relate to recurrence and overall survival (OS). This retrospective single-center study addresses this gap in patients with locally advanced, resectable gastroesophageal adenocarcinoma treated with neoadjuvant or perioperative systemic therapy followed by surgery.

## Materials and methods

### Cohort definition and patient selection

This retrospective study initially identified 692 consecutive adult patients diagnosed with locally advanced, resectable gastroesophageal adenocarcinoma at the Medical University of Vienna between 1 January 2006 and 31 December 2024. Of these, 394 patients received neoadjuvant or perioperative systemic therapy followed by surgery. TRG analyses were restricted to 175 patients with available TRG assessment in the resection specimen. The start date of 2006 was chosen because this marked the publication of the first pivotal study demonstrating a survival benefit of neoadjuvant chemotherapy in this disease setting, leading to its implementation in clinical practice.^17^

Eligible patients had locally advanced, resectable disease at diagnosis. This included tumors classified as clinical stage II, stage III, or stage II-III in cases in which precise differentiation between stages II and III was not feasible. In some patients, endosonography and/or laparoscopic investigation of the peritoneal cavity was omitted due to time constraints or clinical considerations; therefore, staging was based on the best available radiological assessment. Tumor staging was carried out according to the Union for International Cancer Control, Tumor-Node-Metastatsis (UICC TNM) classification. To ensure consistency across the study period, all patients were retrospectively reclassified according to the eighth edition of the UICC TNM staging system. Initial staging was carried out using computed tomography imaging, and histopathological confirmation of adenocarcinoma was mandatory in all cases. TRG was assessed according to the Mandard and Becker classification systems as originally described in the respective publications.^6^^,^^7^ All pathological evaluations were carried out or reviewed by experienced gastrointestinal pathologists to ensure diagnostic accuracy. Tumor regression grading was assessed according to routine pathological practice at the time of resection. Consequently, Mandard and Becker TRG were not both available for all cases included in this retrospective cohort.

Treatment decisions were made within a multidisciplinary tumor board and were guided by contemporary clinical standards applicable at the time of diagnosis. Patients received neoadjuvant or perioperative therapy followed by surgery according to institutional protocols and evolving guideline recommendations during the study period.

### Data collection

Clinical and pathological data, including patient demographics, tumor characteristics, treatment details, and follow-up information, were extracted from structured electronic medical records.

Disease-free survival (DFS) was defined as the interval from tumor resection to the first documented local and/or distant recurrence. Patients without recurrence were censored at the date of last follow-up or death. OS was calculated from the date of initial cancer diagnosis to death from any cause or last follow-up.

Survival follow-up was conducted until 29 December 2025. For patients without a recorded date of death by this cut-off, survival times were censored at the date of last documented contact. Mortality data were obtained from hospital records and cross-checked, when available, with the Austrian national registry (Statistik Austria, Vienna, Austria) data to ensure completeness.

### Statistical analysis

Associations between clinicopathological characteristics and treatment-related variables were assessed using chi-square tests or Fisher’s exact tests, as appropriate. Survival probabilities were estimated using the Kaplan–Meier method and compared using the log-rank test. Univariable analyses were carried out to identify potential prognostic factors. Multivariable survival analysis using Cox proportional hazards regression was explored.

All statistical tests were two-sided, and a *P* < 0.05 was considered statistically significant. As this study was exploratory and hypothesis-generating, no adjustments for multiple testing were applied.^18^ Statistical analyses were conducted using R software (R Foundation for Statistical Computing, Vienna, Austria).

## Results

### Overall cohort and changes throughout the years

In total, 692 patients with locally advanced, resectable disease were included in this analysis [76% male, median age 66; tumor location: esophagus 17%, gastroesophageal junction (GEJ) 44%, stomach 39%; stage: II 26%, II-III 25%, and III 49%]. Among these patients, 123 (18%) received perioperative therapy, 271 (39%) neoadjuvant therapy only, and 45 (7%) adjuvant therapy only. Of the 394 patients (57%) who received a preoperative systemic therapy (either neoadjuvant or perioperative) with or without radiation treatment, 175 (44%) had a recorded TRG. Within these 175 patients, 52 (13%) had a Mandard regression score documented, 6 (2%) had a Becker regression score only, and 117 (30%) had both Mandard and Becker scores recorded.

With respect to the year of diagnosis, the number of resectable patients receiving neoadjuvant or perioperative chemotherapy as well as the number of patients assigned a TRG status increased over time (Table 1).

**Table 1:** Temporal trends in treatment strategy and tumor regression grading

Year of diagnosis	Overall cohort of resectable patients (*n* = 692)	Patients who received neoadjuvant/perioperative systemic therapy (*n* = 394)	Patients with available TRG status (*n* = 175)
2005-2009	184	86 (47%)	5 (6%)
2010-2014	191	106 (55%)	40 (38%)
2015-2019	176	99 (56%)	62 (63%)
2020-2024	141	103 (73%)	68 (66%)

Distribution of patients with resectable, locally advanced disease according to year of diagnosis. The table summarizes the proportion of patients receiving neoadjuvant or perioperative systemic therapy and the proportion of patients with documented tumor regression grade (TRG) over time.

### Patient and tumor characteristics of patients with TRG status

Among the 175 patients with available TRG data, 81% were male and the median age was 65 years. Tumors were located at the GEJ in 53% of cases. Further patient and tumor characteristics are summarized in Supplementary Tables S1 and S2 (available at https://doi.org/10.1016/j.esmogo.2026.100408). Detailed information on therapeutic strategies and systemic regimens is provided in Supplementary Table S3 (available at https://doi.org/10.1016/j.esmogo.2026.100408). In this cohort, 37 patients (21%) received first-line systemic therapy at the time of recurrence.

Regarding tumor regression, in the 123 patients assessed according to Becker, 53 (43%) were classified as TRG 3 (>50% residual tumor). According to the Mandard classification, available in 169 patients, more than half were classified as TRG 3 (residual cancer cells outgrown by fibrosis) or TRG 4 (residual cancer cells outgrowing fibrosis). With respect to pathological TNM (tumor–node–metastasis) staging, missing data varied among variables; ypT was the most frequently recorded parameter with only three missing values, whereas ypM was rarely recorded as it is usually not reported at all if negative. Detailed distributions of TRG according to Becker and Mandard as well as pathological TNM staging are shown in Table 2.

**Table 2:** Distribution of TRG and pathological staging

Characteristic	*n* = 175^a^
TRG (Becker)	
1a (0% residual tumor)	24 (20)
1b (<10% residual tumor)	20 (16)
2 (10%-50% residual tumor)	26 (21)
3 (>50% residual tumor)	53 (43)
Missing	52
TRG (Mandard)	
1 (no residual cancer cells)	28 (17)
2 (rare residual cancer cells)	31 (18)
3 (more residual cancer cells, but outgrown by fibrosis)	41 (24)
4 (residual cancer cells outgrowing fibrosis)	46 (27)
5 (absence of regressive changes)	23 (14)
Missing	6
ypT	
0	27 (16)
1	24 (14)
2	33 (19)
3	81 (47)
4	7 (4)
Missing	3
ypN	
0	93 (55)
1	28 (17)
2	24 (14)
3	24 (14)
Missing	6
ypM	
0	19 (100)
Missing	156
ypL	
0	82 (57)
1	62 (43)
Missing	31
ypV	
0	136 (87)
1	20 (13)
Missing	19
ypPn	
0	63 (59)
1	43 (41)
Missing	69
R	
0	146 (94)
1	10 (6)
Missing	19

Distribution of TRG according to Becker and Mandard classifications and pathological TNM (ypTNM) parameters among patients with available TRG data. Missing values are indicated for each variable.

TRG, tumor regression grade.

a Values are presented as *n* (%).

### Association of TRG with patient, tumor, and treatment characteristics

In the analysis of associations between TRG and clinicopathological characteristics, Becker TRG was significantly associated with several parameters including age (*P* = 0.032; higher regression scores in older patients) and MMR status (*P* < 0.001; lower regression scores in patients with deficient MMR). A strong association was observed between Becker and Mandard TRG (*P* < 0.001; lower Becker scores corresponding to lower Mandard scores and higher Becker scores corresponding to higher Mandard scores). Furthermore, Becker TRG was significantly associated with ypT (*P* < 0.001; lower regression scores in lower pT categories), ypN (*P* = 0.005; lower regression scores in lower pN categories), ypL (*P* < 0.001; lower regression scores in the absence of lymphatic invasion), ypV (*P* = 0.047; lower regression scores in the absence of venous invasion), and ypPn (*P* = 0.006; lower regression scores in the absence of perineural invasion). In addition, there was an association with neoadjuvant therapy (*P* < 0.001, three immunotherapy-only patients having TRG 1a).

No significant association was found with resection margin status R (*P* = 0.4; no difference in regression scores according to margin status) or any other patient or tumor characteristics. These results are presented in Supplementary Table S4 (available at https://doi.org/10.1016/j.esmogo.2026.100408).

Similarly, Mandard TRG was significantly associated with Becker TRG (*P* < 0.001), ypT (*P* < 0.001; lower Mandard scores in lower pT categories), ypN (*P* = 0.007; lower Mandard scores in lower pN categories), ypL (*P* < 0.001; lower Mandard scores in the absence of lymphatic invasion), ypV (*P* = 0.027; lower Mandard scores in the absence of venous invasion), and ypPn (*P* < 0.001; lower Mandard scores in the absence of perineural invasion), and neoadjuvant therapy (*P* = 0.001, three immunotherapy-only patients having TRG 1), but not with resection margin status R (*P* = 0.5) or other patient or tumor characteristics. Detailed results are shown in Supplementary Table S5 (available at https://doi.org/10.1016/j.esmogo.2026.100408).

Concerning the 10 patients who were MMR deficient, 3 received combination immunotherapy with nivolumab/ipilimumab and all 3 had TRG 1, whereas with 4 patients receiving immunochemotherapy, results were more diverse, with 1 TRG 1, 2 TRG 3, and 1 TRG 5 according to Mandard.

### DFS and OS

At the time of data cut-off on 29 December 2025, 70 patients (40%) had experienced recurrence and 72 patients (41%) had died. Median DFS was 44.8 months [95% confidence interval (CI) 30.7-108], and median OS was 58.2 months (95% CI 51.2-108.0). The median follow-up time was 50.1 months (95% CI 39.1-62.7).

#### DFS

In the univariable analysis of DFS, Mandard TRG (*P* = 0.0062), ypT (*P* < 0.0001), ypN (*P* < 0.0001), ypV (*P* = 0.0019), ypL (*P* = 0.022), ypPn (*P* = 0.0022), stage (*P* = 0.033), tumor location (*P* = 0.026), and MMR status (*P* = 0.012) were statistically significantly associated with DFS. Detailed results are presented in Table 3.

**Table 3:** Univariable analysis of DFS

Variable	Category	*n*	Events	Median DFS (months)	95% CI	*P* value
Mandard TRG	TRG 1	28	9	112.1	40.7-NA	0.0063
	TRG 2	31	12	NA	22.9-NA	
	TRG 3	41	20	31.9	19.6-NA	
	TRG 4	46	22	46.7	22.0-NA	
	TRG 5	23	15	9.8	5.4-NA	
ypT (tumor depth)	ypT0	27	9	112.1	40.7-NA	<0.0001
	ypT1	24	7	NA	43.2-NA	
	ypT2	33	9	NA	108.4-NA	
	ypT3	81	51	22.0	15.8-36	
	ypT4	7	5	10.5	5.4-NA	
ypN (nodal status)	ypN0	93	29	NA	70.2-NA	<0.0001
	ypN1	28	18	28.6	19.6-NA	
	ypN2	24	16	17.7	15.0-NA	
	ypN3	24	17	14.6	7.9-NA	
ypV (venous invasion)	ypV0	136	59	49.0	34.7-NA	0.0019
	ypV1	20	15	21.7	8.3-NA	
ypL (lymphatic invasion)	ypL0	82	30	70.2	43.2-NA	0.022
	ypL1	62	35	25.9	15.6-108	
ypPn (perineural invasion)	ypPn0	63	22	70.2	40.7-NA	0.0022
	ypPn1	43	28	22.0	15.6-49	
Stage	Stage II	28	6	NA	43.2-NA	0.033
	Stage II-III	60	32	32.3	14.6-NA	
	Stage III	87	44	36.1	25.9-108	
Tumor location	GEJ	93	41	85.8	25.9-NA	0.026
	Stomach	47	20	68.4	45.4-NA	
	Esophagus	35	21	27.9	13.4-40.7	
MMR status	Deficient	10	0	NA	NA-NA	0.012
	Proficient	98	46	30.7	22.9-NA	

Results of univariable log-rank analyses evaluating associations between clinicopathological characteristics and DFS. Median time in months, 95% CI, and *P* values are shown.

CI, confidence interval; DFS, disease-free survival; GEJ, gastroesophageal junction; MMR, mismatch repair; TRG, tumor regression grade.

Age (*P* = 0.84), alcohol consumption (*P* = 0.8), body mass index (BMI) (*P* = 0.21), HER2 status (*P* = 0.69), PD-L1 status (*P* = 0.61), R (*P* = 0.074), sex (*P* = 0.13), signet ring cell histology (*P* = 0.35), smoking (*P* = 0.19), treatment strategy (neoadjuvant only versus perioperative, *P* = 0.14), neoadjuvant regimen (*P*=0.19), and Becker TRG (*P* = 0.23) were not significantly associated with DFS.

Supplementary Figure S1 (available at https://doi.org/10.1016/j.esmogo.2026.100408) shows Kaplan–Meier curves for DFS stratified by TRG according to the Becker and Mandard classifications. Supplementary Figure S2 (available at https://doi.org/10.1016/j.esmogo.2026.100408) presents Kaplan–Meier curves for DFS according to the ypTNM classification.

#### OS

In the univariable analysis of OS, significant associations were observed for Mandard TRG (*P* = 0.0015), ypT (*P* < 0.0001), ypN (*P* < 0.0001), ypV (*P* = 0.026), ypL (*P* = 0.015), ypPn (*P* = 0.0057), R (*P* = 0.016), sex (*P* = 0.031), smoking status (*P* = 0.034), MMR status (*P* = 0.042), and recurrence (*P* < 0.0001). Detailed results are presented in Table 4.

**Table 4:** Univariable analysis of OS

Variable	Category	*n*	Events	Median OS (months)	95% CI	*P* value
Mandard TRG	TRG 1	28	8	85.8	51.3-NA	0.0015
	TRG 2	31	11	NA	42.4-NA	
	TRG 3	41	14	44.2	38.9-NA	
	TRG 4	46	20	59.3	51.5-NA	
	TRG 5	23	15	18.1	13.4-NA	
ypT (tumor depth)	ypT0	27	8	85.8	51.3-NA	<0.0001
	ypT1	24	5	NA	59.3-NA	
	ypT2	33	9	NA	108.4-NA	
	ypT3	79	43	43.1	27.8-66.4	
	ypT4	7	5	16.9	16.5-NA	
ypN (nodal status)	ypN0	93	24	112.1	66.4-NA	<0.0001
	ypN1	28	14	51.3	23.9-NA	
	ypN2	24	15	27.8	21.1-NA	
	ypN3	22	16	32.0	23.2-55.9	
ypL (lymphatic invasion)	ypL0	82	25	66.4	53.5-NA	0.015
	ypL1	60	32	42.4	28.6-101	
ypV (venous invasion)	ypV0	135	52	58.2	47.8-NA	0.026
	ypV1	19	12	31.5	17.1-NA	
ypPn (perineural invasion)	ypPn0	62	19	59.3	51.3-NA	0.0057
	ypPn1	42	24	31.5	23.2-101	
ypR (resection margin)	ypR0	144	58	66.4	51.5-NA	0.016
	ypR1	10	6	23.9	17.4-NA	
Sex	Male	140	62	53.4	43.2-78.9	0.031
	Female	33	9	NA	101.2-NA	
Smoking status	No	60	16	NA	68.4-NA	0.034
	Abuse	93	44	51.3	43.1-108	
MMR status	Deficient	10	0	NA	NA-NA	0.042
	Proficient	97	38	55.9	42.4-NA	
Recurrence	No	104	12	NA	NA-NA	<0.0001
	Yes	69	59	27.4	21.7-42.4	

Results of univariable log-rank analyses evaluating associations between clinicopathological characteristics and OS. Median time in months, 95% CI, and *P* values are shown.

CI, confidence interval; MMR, mismatch repair; OS, overall survival; TRG, tumor regression grade.

In contrast, age (*P* = 0.63), alcohol consumption (*P* = 0.56), BMI (*P* = 0.37), HER2 status (*P* = 0.55), PD-L1 status (*P* = 0.6), signet ring cell histology (*P* = 0.12), overall stage (*P* = 0.14), treatment strategy (*P* = 0.1), neoadjuvant regimen (*P* = 0.46), Becker TRG (*P* = 0.31), and tumor location (*P* = 0.12) were not significantly associated with OS.

**Figure 1:**
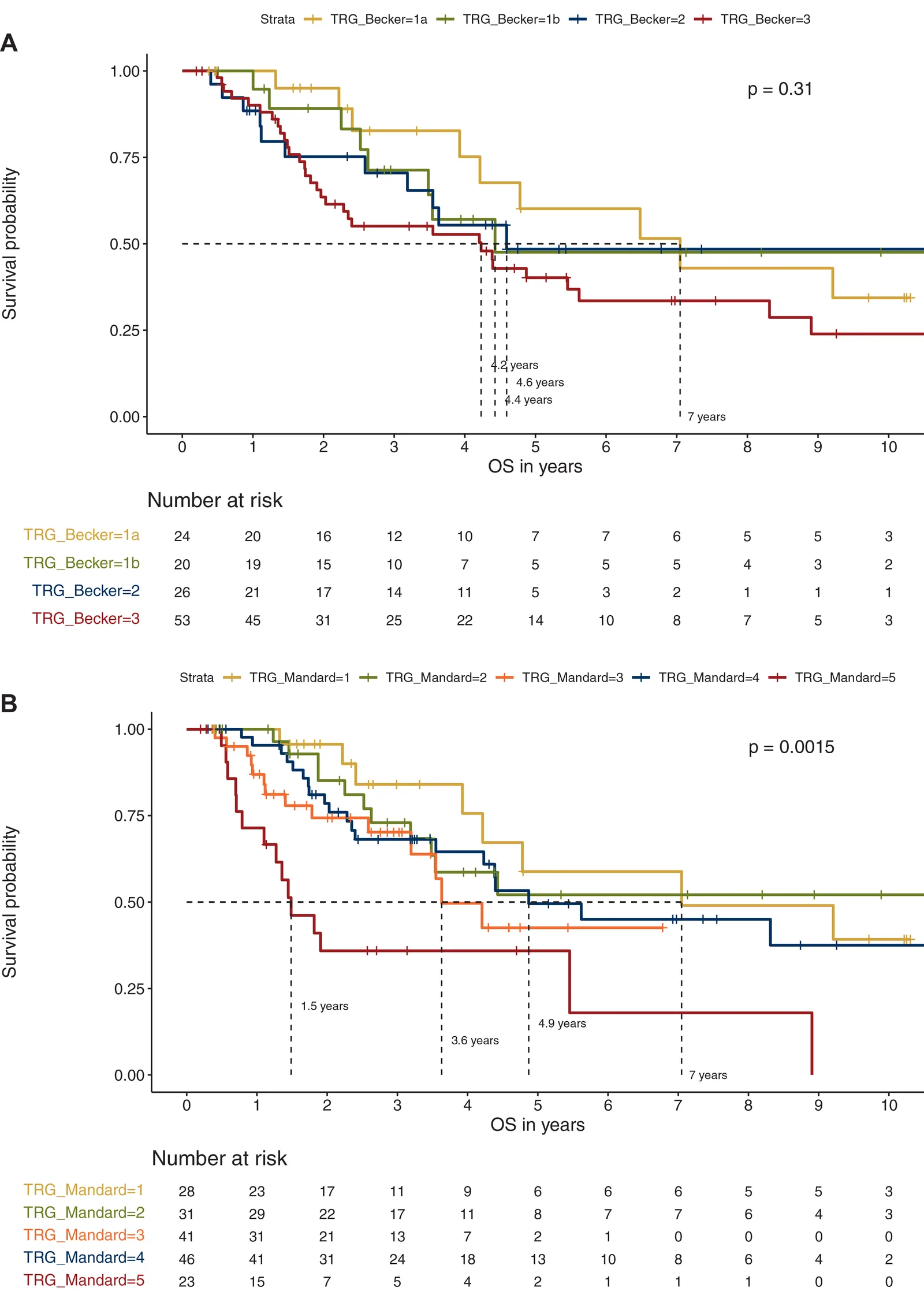
**Overall survival (OS) according to tumor regression grade (TRG).** Kaplan–Meier curves for OS stratified by TRG according to the Becker and Mandard classifications. *P* values were calculated using the log-rank test.

**Figure 2:**
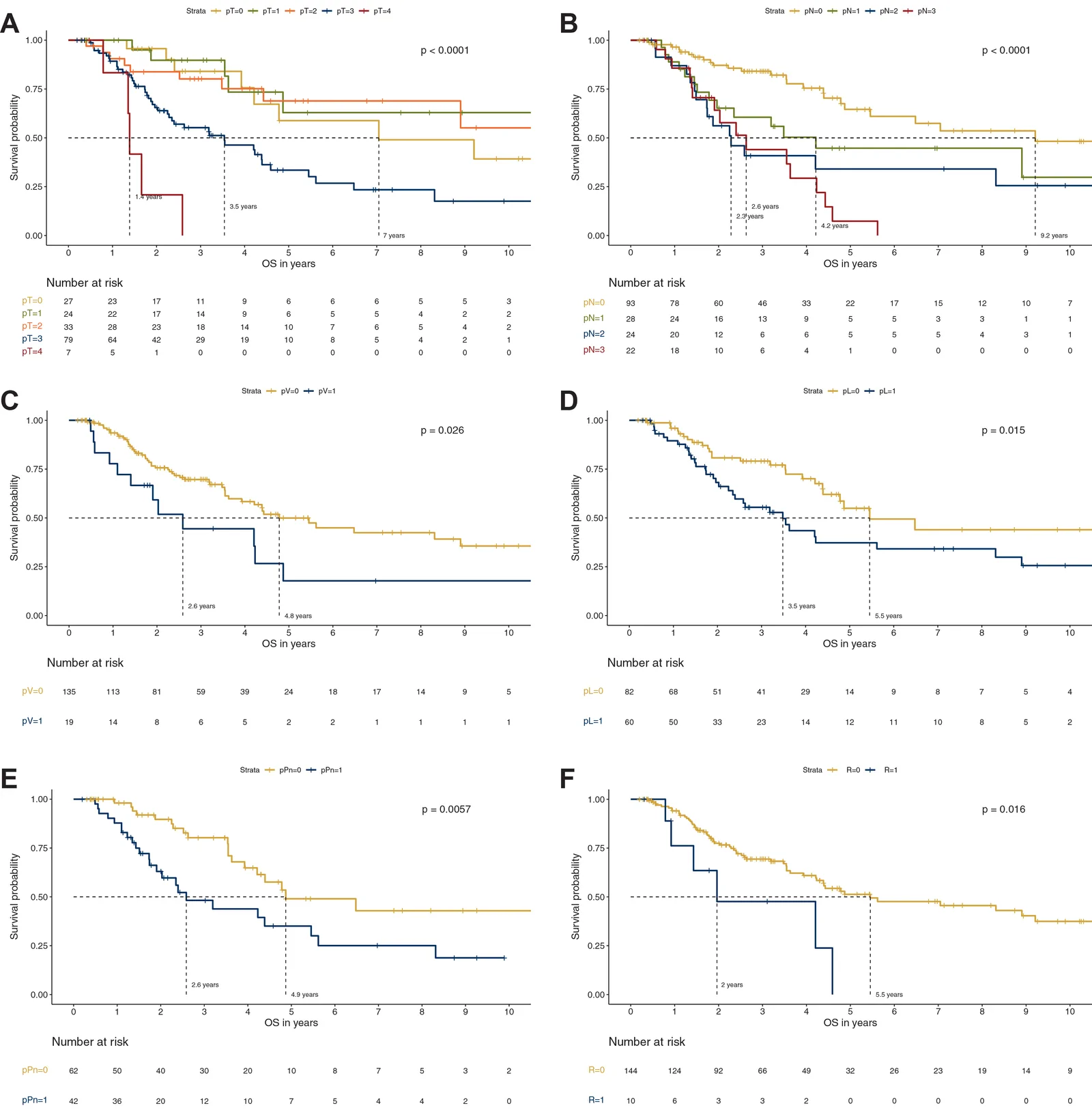
**Overall survival (OS) according to ypTNM classification.** Kaplan–Meier curves for OS stratified by pathological TNM (ypTNM) stage. *P* values were calculated using the log-rank test.

Figure 1 illustrates OS stratified by TRG according to the Becker and Mandard classifications. Figure 2 shows OS according to the ypTNM classification.

#### Subgroup analyses according to neoadjuvant treatment strategy

To address potential treatment heterogeneity, additional subgroup analyses were carried out according to neoadjuvant treatment strategy. In patients treated with FLOT-based chemotherapy, Mandard TRG remained significantly associated with both DFS (*P* = 0.026) and OS (*P* = 0.0037), whereas Becker TRG was not associated with either DFS (*P* = 0.53) or OS (*P* = 0.31). Among patients receiving non-FLOT chemotherapy, Mandard TRG remained associated with OS (*P* = 0.048) and showed a borderline association with DFS (*P* = 0.058), whereas Becker TRG was not associated with DFS (*P* = 0.34) or OS (*P* = 0.32). In contrast, within the radiochemotherapy subgroup, both Becker TRG (DFS, *P* = 0.00082; OS, *P* = 0.0077) and Mandard TRG (DFS, *P* = 0.00019; OS, *P* < 0.0001) were significantly associated with survival outcomes.

To further explore whether post-operative treatment influenced the prognostic value of TRG, additional subgroup analyses were carried out according to treatment strategy (neoadjuvant versus perioperative). Among patients receiving neoadjuvant treatment only, Mandard TRG remained significantly associated with DFS (*P* = 0.00069) and OS (*P* = 0.00048), whereas Becker TRG was not associated with either DFS (*P* = 0.41) or OS (*P* = 0.48). In contrast, neither Mandard nor Becker TRG was significantly associated with DFS (Mandard, *P* = 0.53; Becker, *P* = 0.37) or OS (Mandard, *P* = 0.60; Becker, *P* = 0.46) in patients receiving perioperative treatment.

#### Multivariable analyses

Multivariable models were explored. However, given the limited number of events relative to the number of candidate predictors and the substantial proportion of missing data, the models were considered at high risk of overfitting and yielded unstable estimates. Therefore, multivariable results are not presented.

## Discussion

In this retrospective single-center study, evaluation of TRG in locally advanced gastroesophageal adenocarcinoma increased substantially over time, paralleling the broader implementation of perioperative therapy and heightened awareness of pathological response as a potential prognostic marker. However, our analysis shows that pathological tumor regression after neoadjuvant therapy is highly heterogeneous, with a predominance of poor responders by both Mandard and Becker TRG. In line with previous reports, lower TRG scores were closely associated with more favorable ypT and ypN categories and the absence of lymphatic, venous, and perineural invasion, underscoring that TRG might reflect both treatment effect and underlying tumor biology.^9^^,^^19^^,^^20^ The strong concordance observed between Mandard and Becker classifications further supports that both systems capture tumor response.^5^ However, their different analysis methods (fibrosis–tumor balance^6^ versus percentage of residual viable tumor^7^) may account for differences in prognostic discrimination, as only Mandard TRG was significantly associated with DFS and OS in our cohort. Thus, the choice of grading system and category definitions may influence the apparent prognostic value of TRG and further efforts toward improvement of TRG assessments are needed, which is in line with recent findings from other cohorts.^9^^,^^10^^,^^21^, ^22^, ^23^

The additional subgroup analyses addressing treatment heterogeneity further support these observations. Among patients treated with chemotherapy, Mandard TRG remained associated with survival in both the FLOT-based and non-FLOT cohorts, whereas Becker TRG was not associated with either DFS or OS. These findings suggest that the prognostic performance of Mandard TRG is not solely driven by the inclusion of different chemotherapy regimens. In contrast, both Mandard and Becker TRG were associated with survival in the radiochemotherapy subgroup. Additional exploratory analyses according to treatment strategy demonstrated that the prognostic association of Mandard TRG was maintained in patients receiving neoadjuvant treatment only but was not observed in the perioperative subgroup. Given the limited sample size and the distinct clinical characteristics of these patients, these findings should be interpreted cautiously and warrant validation in larger, more homogeneous cohorts.

Importantly, whether Mandard TRG in our cohort truly reflects a robust prognosticator remains uncertain, as classical pathological factors (ypT, ypN, ypV, ypPn, and R) remained the strongest prognostic determinants for both DFS and OS. This underscores that TRG may largely operate through its association with pathological downstaging rather than representing an entirely independent prognostic marker. Notably, the pronounced prognostic impact of TRG reported in some prior studies may therefore partly reflect incomplete adjustment for established pathological risk factors. Analyses that do not incorporate ypT, ypN, vascular invasion, and margin status may overestimate the independent effect of regression grading.^19^^,^^24^ Unfortunately, in our cohort, multivariable analyses were limited by event numbers and missing data, so an independent prognostic role of TRG beyond ypTNM can neither be definitively excluded nor robustly demonstrated. Thus, future studies will require adequately powered multivariable models to clarify whether TRG provides true prognostic value sufficient to influence post-operative decision-making.

Unlike previous studies that primarily focused on comparing TRG classification systems,^5^^,^^21^^,^^25^ our study integrates TRG assessment with pathological risk factors, molecular biomarkers, and treatment heterogeneity across a contemporary real-world cohort. Thus, a major strength of our study is that biomarker testing, including HER2, MMR, and PD-L1, in localized gastroesophageal adenocarcinoma has recently been routinely implemented at our center, allowing exploratory analyses of molecular features in a real-world perioperative population. The observed associations of TRG with MMR and PD-L1 status are hypothesis-generating and suggest that molecular and immunological tumor features may modulate treatment sensitivity and regression patterns because MMR-deficient tumors are characterized by high mutational burden and enhanced immunogenicity, potentially rendering them more susceptible to immune-mediated cytotoxicity.^26^ The few MMR-deficient patients in our cohort seemed to have pCRs with doublet immunotherapy, which is in line with results from ongoing trials.^27^ However, given that these patients predominantly received immunotherapy-based regimens, it remains unclear to what extent the observed responses are driven by the underlying molecular phenotype itself versus the specific treatment applied.

In line with this, another study group reported no substantial differences in tumor regression between patients with MMR-deficient and MMR-proficient tumors treated with chemotherapy alone.^28^ Taken together, these findings highlight that disentangling the independent effect of molecular alterations from treatment-related effects is challenging in retrospective cohorts with heterogeneous therapies. As the number of patients in our cohort is very limited, this should be evaluated in larger populations. These preliminary findings nevertheless support the concept of biomarker-driven perioperative strategies and raise the hypothesis that TRG patterns may differ fundamentally between chemotherapy-only and chemo-immunotherapy regimens.

As the perioperative therapeutic landscape is now shifting toward chemo-immunotherapy, with the addition of durvalumab to FLOT as a new standard of care in fit patients with resectable gastric and GEJ adenocarcinoma,^16^ pathological response and TRG may gain further relevance as integrated readouts of combined chemo-immunotherapy efficacy.^13^^,^^14^ In this context, TRG could evolve from a purely descriptive histopathological parameter to a surrogate marker of composite chemo-immunological treatment sensitivity. However, it remains uncertain whether conventional TRG systems, originally developed in the context of cytotoxic chemotherapy, are sufficient to capture response patterns after immunotherapy, such as dense immune cell infiltration or tumor–immune interactions. Therefore, adapted immune-related pathological response criteria may be required to more accurately assess treatment response in this setting.

Further strengths and limitations of this analysis need to be addressed. This study provides a large, long-term, real-world analysis of TRG patterns, pathological risk factors, and outcomes in localized gastroesophageal adenocarcinoma. By spanning nearly two decades of evolving systemic therapy, our cohort captures the transition from early perioperative chemotherapy approaches to contemporary multimodal strategies, thereby offering a reference framework against which outcomes in the upcoming immunotherapy era can be compared. However, these substantial changes in treatment strategies and the resulting heterogeneity of the cohort represent an additional limitation. Although we carried out subgroup analyses according to treatment regimen to address this issue, several treatment-related variables were not consistently available, as expected in a retrospective cohort. In particular, the number of completed neoadjuvant and post-operative treatment cycles could not be analyzed, although treatment intensity may have influenced pathological response and survival outcomes.

Further limitations encompass the retrospective design with potential selection bias and missing values, as well as the limited number of events inhibiting stable multivariable modeling. As this study was designed as an exploratory, hypothesis-generating analysis, no adjustment for multiple testing was carried out. Consequently, the reported *P* values should be interpreted cautiously, and the observed associations require validation in independent prospective cohorts.

### Conclusion

Although Mandard TRG was associated with OS in our cohort, this effect may largely be driven by its close association with established pathological risk factors such as ypT and ypN, as well as underlying molecular targets including MMR and PD-L1 status and respective treatment strategies. Thus, although TRG may contribute to risk stratification, its independent clinical value remains uncertain. Prospective studies integrating standardized TRG assessment with molecular biomarkers and immune features are therefore essential to clarify its role in guiding post-operative treatment and surveillance strategies, particularly in the rapidly evolving era of perioperative chemo-immunotherapy.
