# Supplementary material for: Tumor regression grades in locally advanced gastroesophageal adenocarcinoma: associations with survival, pathological risk factors, and molecular biomarkers in a 19-year real-world cohort
Source: ESMO Gastrointest Oncol. 2026 Sep 2;13(Pt B):100408. doi: 10.1016/j.esmogo.2026.100408 (PMC13571382; doi:10.1016/j.esmogo.2026.100408)
Supplement: Supplementary file 1 [file mmc1.pdf]

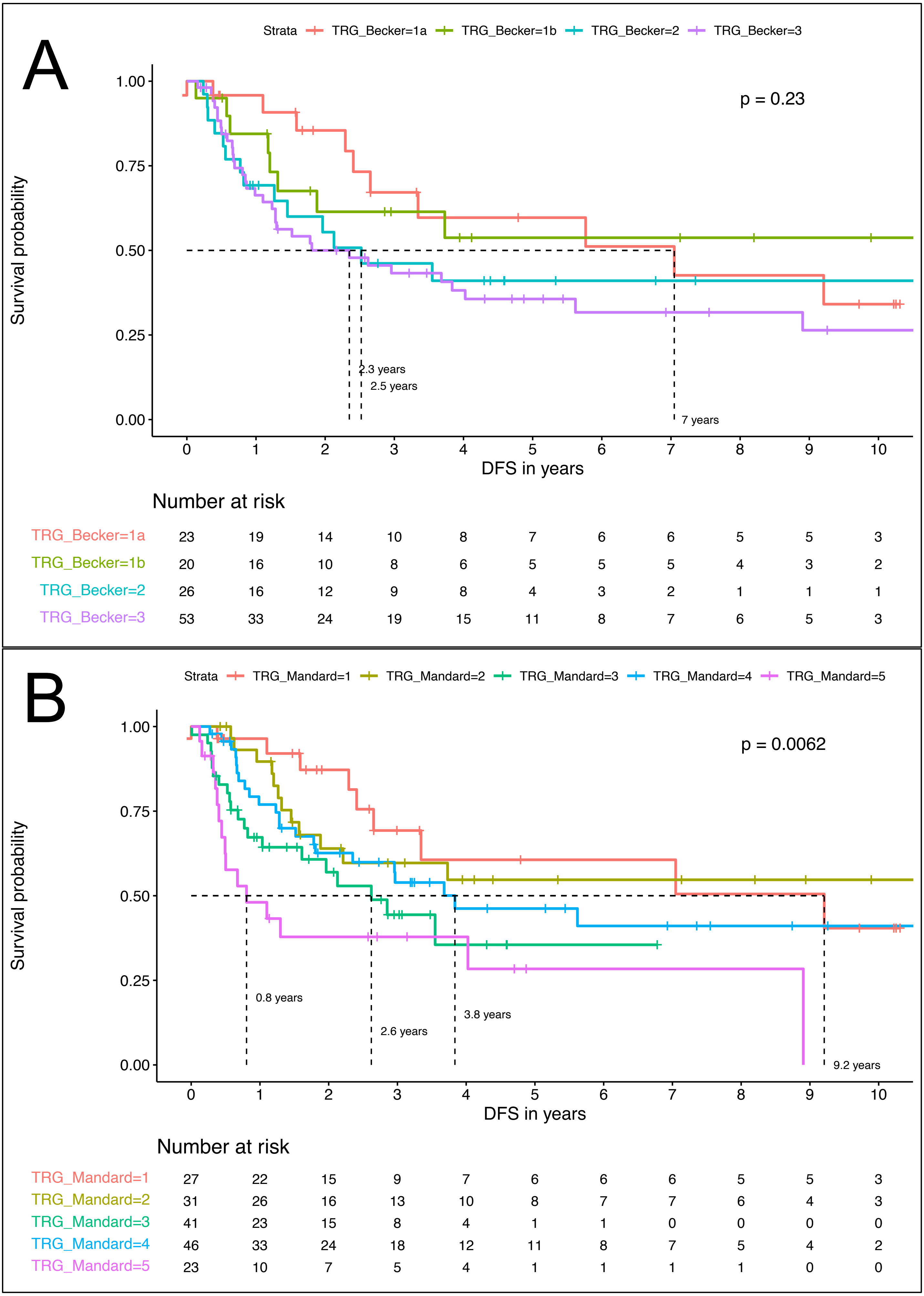
A
Strata
TRG_Becker=1a
TRG_Becker=1b
TRG_Becker=2
TRG_Becker=3
p = 0.23
Survival probability
1.00
0.75
0.50
0.25
0.00
2.3 years
2.5 years
7 years
0 1 2 3 4 5 6 7 8 9 10
DFS in years
Number at risk
TRG_Becker=1a 23 19 14 10 8 7 6 6 5 5 3
TRG_Becker=1b 20 16 10 8 6 5 5 5 4 3 2
TRG_Becker=2 26 16 12 9 8 4 3 2 1 1 1
TRG_Becker=3 53 33 24 19 15 11 8 7 6 5 3
B
Strata
TRG_Mandard=1
TRG_Mandard=2
TRG_Mandard=3
TRG_Mandard=4
TRG_Mandard=5
p = 0.0062
Survival probability
1.00
0.75
0.50
0.25
0.00
0.8 years
2.6 years
3.8 years
9.2 years
0 1 2 3 4 5 6 7 8 9 10
DFS in years
Number at risk
TRG_Mandard=1 27 22 15 9 7 6 6 6 5 5 3
TRG_Mandard=2 31 26 16 13 10 8 7 7 6 4 3
TRG_Mandard=3 41 23 15 8 4 1 1 0 0 0 0
TRG_Mandard=4 46 33 24 18 12 11 8 7 5 4 2
TRG_Mandard=5 23 10 7 5 4 1 1 1 1 0 0
