# Supplementary material for: Tumor regression grades in locally advanced gastroesophageal adenocarcinoma: associations with survival, pathological risk factors, and molecular biomarkers in a 19-year real-world cohort
Source: ESMO Gastrointest Oncol. 2026 Sep 2;13(Pt B):100408. doi: 10.1016/j.esmogo.2026.100408 (PMC13571382; doi:10.1016/j.esmogo.2026.100408)
Supplement: Supplementary file 2 [file mmc2.pdf]

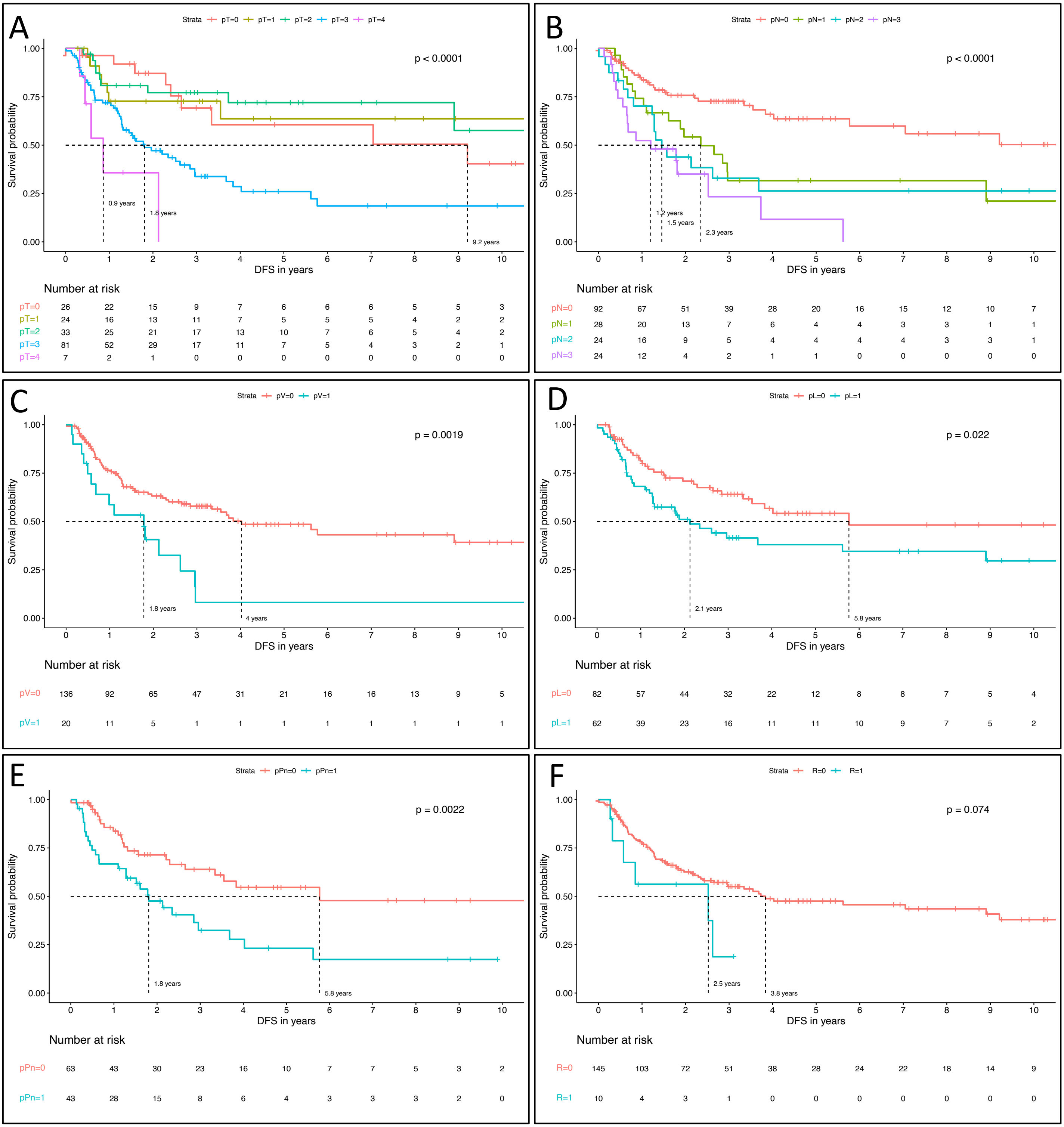
A
Strata pT=0 pT=1 pT=2 pT=3 pT=4
p < 0.0001
Survival probability
DFS in years
0.9 years
1.8 years
9.2 years
Number at risk
pT=0 26 22 15 9 7 6 6 6 5 5 3
pT=1 24 16 13 11 7 5 5 5 4 2 2
pT=2 33 25 21 17 13 10 7 6 5 4 2
pT=3 81 52 29 17 11 7 5 4 3 2 1
pT=4 7 2 1 0 0 0 0 0 0 0 0
B
Strata pN=0 pN=1 pN=2 pN=3
p < 0.0001
Survival probability
DFS in years
1.2 years
1.5 years
2.3 years
Number at risk
pN=0 92 67 51 39 28 20 16 15 12 10 7
pN=1 28 20 13 7 6 4 4 3 3 1 1
pN=2 24 16 9 5 4 4 4 4 3 3 1
pN=3 24 12 4 2 1 1 0 0 0 0 0
C
Strata pV=0 pV=1
p = 0.0019
Survival probability
DFS in years
1.8 years
4 years
Number at risk
pV=0 136 92 65 47 31 21 16 16 13 9 5
pV=1 20 11 5 1 1 1 1 1 1 1 1
D
Strata pL=0 pL=1
p = 0.022
Survival probability
DFS in years
2.1 years
5.8 years
Number at risk
pL=0 82 57 44 32 22 12 8 8 7 5 4
pL=1 62 39 23 16 11 11 10 9 7 5 2
E
Strata pPn=0 pPn=1
p = 0.0022
Survival probability
DFS in years
1.8 years
5.8 years
Number at risk
pPn=0 63 43 30 23 16 10 7 7 5 3 2
pPn=1 43 28 15 8 6 4 3 3 3 2 0
F
Strata R=0 R=1
p = 0.074
Survival probability
DFS in years
2.5 years
3.8 years
Number at risk
R=0 145 103 72 51 38 28 24 22 18 14 9
R=1 10 4 3 1 0 0 0 0 0 0 0
