# Supplementary material for: Tumor regression grades in locally advanced gastroesophageal adenocarcinoma: associations with survival, pathological risk factors, and molecular biomarkers in a 19-year real-world cohort
Source: ESMO Gastrointest Oncol. 2026 Sep 2;13(Pt B):100408. doi: 10.1016/j.esmogo.2026.100408 (PMC13571382; doi:10.1016/j.esmogo.2026.100408)
Supplement: Supplementary file 3 [file mmc3.docx]

| **Characteristic** | **N = 175**^1^ |
| --- | --- |
| **Sex** |  |
| Female | 33 (19%) |
| Male | 142 (81%) |
| **Age** |  |
| Mean (SD) | 63 (11) |
| Median (IQR) | 65 (57, 71) |
| Range | 23, 83 |
|  |  |
| <=45 | 12 (7%) |
| >=65 | 88 (50%) |
| >45 <65 | 75 (43%) |
| **BMI** |  |
| normal weight (18.5–24.9) | 68 (46%) |
| obesity (≥30.0) | 20 (14%) |
| overweight (25.0–29.9) | 57 (39%) |
| underweight (<18.5) | 2 (1%) |
| (Missing) | 28 |
| **Alcohol consumption** |  |
| Alcohol abuse | 18 (12%) |
| Moderate alcohol consumption | 67 (43%) |
| No alcohol consumption | 70 (45%) |
| (Missing) | 20 |
| **Nicotine abuse** |  |
| Abuse | 94 (61%) |
| No | 61 (39%) |
| (Missing) | 20 |
| **Recurrence/Progression** |  |
| No recurrence/progression | 105 (60%) |
| Recurrence/Progression | 70 (40%) |
| **Vital status** |  |
| Alive | 103 (59%) |
| Deceased | 72 (41%) |
| ^1^n (%) | |
