# Supplementary material for: Tumor regression grades in locally advanced gastroesophageal adenocarcinoma: associations with survival, pathological risk factors, and molecular biomarkers in a 19-year real-world cohort
Source: ESMO Gastrointest Oncol. 2026 Sep 2;13(Pt B):100408. doi: 10.1016/j.esmogo.2026.100408 (PMC13571382; doi:10.1016/j.esmogo.2026.100408)
Supplement: Supplementary file 4 [file mmc4.docx]

| **Characteristic** | **N = 175**^1^ |
| --- | --- |
| **Tumor location** |  |
| Esophagus | 35 (20%) |
| GEJ | 93 (53%) |
| Stomach | 47 (27%) |
| **Stage** |  |
| 2 | 28 (16%) |
| 2-3 | 60 (34%) |
| 3 | 87 (50%) |
| **Signet ring cells** |  |
| negative | 112 (72%) |
| positive | 43 (28%) |
| (Missing) | 20 |
| **MMR** |  |
| deficient | 10 (9%) |
| proficient | 98 (91%) |
| (Missing) | 67 |
| **PD-L1** |  |
| negative | 9 (12%) |
| positive | 69 (88%) |
| (Missing) | 97 |
| **HER2** |  |
| negative | 118 (80%) |
| positive | 29 (20%) |
| (Missing) | 28 |
| ^1^n (%) | |
