# Supplementary material for: Tumor regression grades in locally advanced gastroesophageal adenocarcinoma: associations with survival, pathological risk factors, and molecular biomarkers in a 19-year real-world cohort
Source: ESMO Gastrointest Oncol. 2026 Sep 2;13(Pt B):100408. doi: 10.1016/j.esmogo.2026.100408 (PMC13571382; doi:10.1016/j.esmogo.2026.100408)
Supplement: Supplementary file 5 [file mmc5.docx]

| **Characteristic** | **N = 175**^1^ |
| --- | --- |
| **Surgery** | 175 (100%) |
| **Treatment strategy** |  |
| Neoadjuvant | 112 (64%) |
| Perioperative (neoadjuvant + adjuvant) | 63 (36%) |
| **Neoadjuvant therapy** |  |
| Chemo (FLOT) | 70 (40%) |
| Chemo (not FLOT) | 56 (32%) |
| Chemo+Anti-HER2 | 10 (6%) |
| Chemo+Anti-PD-L1 | 6 (3%) |
| Immunotherapy | 3 (2%) |
| Radiochemotherapy | 25 (14%) |
| unknown | 5 (3%) |
| **Adjuvant therapy** |  |
| Chemo (FLOT) | 28 (44%) |
| Chemo (not FLOT) | 20 (32%) |
| Chemo+Anti-HER2 | 6 (10%) |
| Chemo+Anti-PD-L1 | 4 (6%) |
| Immunotherapy | 2 (3%) |
| Radiochemotherapy | 3 (5%) |
| **Neoadjuvant regimen** |  |
| 5FU mono | 1 (1%) |
| Carboplatin/Paclitaxel | 1 (1%) |
| Cisplatin/5FU | 1 (1%) |
| Cisplatin/5FU/Trastuzumab (TOGA) | 2 (1%) |
| Docetaxel mono | 2 (1%) |
| Docetaxel/Cisplatin/5FU (DCF) | 5 (3%) |
| Docetaxel/Cisplatin/Capecitabin (DCX) | 1 (1%) |
| Epirubicin/Capecitabine | 1 (1%) |
| Epirubicin/Cisplatin/5FU (ECF) | 1 (1%) |
| Epirubicin/Oxaliplatin/Capecitabin (EOX) | 18 (10%) |
| Nivolumb/Ipilimumab | 3 (2%) |
| Oxaliplatin/5FU (FLOT without Docetaxel) | 2 (1%) |
| Oxaliplatin/5FU (FOLFOX) | 9 (5%) |
| Oxaliplatin/Capecitabin (XELOX) | 12 (7%) |
| Oxaliplatin/Docetaxel/5FU (FLOT) | 70 (40%) |
| Oxaliplatin/Docetaxel/5FU/Atezolizumab (FLOT+Atezolizumab) | 4 (2%) |
| Oxaliplatin/Docetaxel/5FU/Nivolumab (FLOT+Nivolumab) | 1 (1%) |
| Oxaliplatin/Docetaxel/5FU/Pembrolizumab (FLOT+Pembrolizumab) | 1 (1%) |
| Oxaliplatin/Docetaxel/5FU/Trastuzumab (FLOT+Trastuzumab) | 8 (5%) |
| Oxaliplatin/Raltitrexed (Oxaliplatin+Tomudex) | 1 (1%) |
| Paclitaxel mono | 1 (1%) |
| RADIATIO + Carboplatin/Paclitaxel (CROSS) | 24 (14%) |
| RADIATIO + Cisplatin/Docetaxel | 1 (1%) |
| unknown | 5 (3%) |
| **Adjuvant Regimen** |  |
| Carboplatin/Paclitaxel | 1 (2%) |
| Docetaxel/Cisplatin/5FU (DCF) | 4 (6%) |
| Epirubicin/Oxaliplatin/Capecitabin (EOX) | 2 (3%) |
| Nivolumab mono | 1 (2%) |
| Nivolumb/Ipilimumab | 1 (2%) |
| Oxaliplatin/5FU (FLOT without Docetaxel) | 4 (6%) |
| Oxaliplatin/5FU (FOLFOX) | 5 (8%) |
| Oxaliplatin/5FU/Trastuzumab (FOLFOX+Trastuzumab) | 1 (2%) |
| Oxaliplatin/Capecitabin (XELOX) | 4 (6%) |
| Oxaliplatin/Docetaxel/5FU (FLOT) | 28 (44%) |
| Oxaliplatin/Docetaxel/5FU/Atezolizumab (FLOT+Atezolizumab) | 2 (3%) |
| Oxaliplatin/Docetaxel/5FU/Pembrolizumab (FLOT+Pembrolizumab) | 2 (3%) |
| Oxaliplatin/Docetaxel/5FU/Trastuzumab (FLOT+Trastuzumab) | 5 (8%) |
| RADIATIO + 5-FU mono | 1 (2%) |
| RADIATIO + Capecitabine mono (Xeloda) | 1 (2%) |
| RADIATIO + Carboplatin/Paclitaxel (CROSS) | 1 (2%) |
|  |  |
| ^1^n (%) | |
