# Supplementary material for: Tumor regression grades in locally advanced gastroesophageal adenocarcinoma: associations with survival, pathological risk factors, and molecular biomarkers in a 19-year real-world cohort
Source: ESMO Gastrointest Oncol. 2026 Sep 2;13(Pt B):100408. doi: 10.1016/j.esmogo.2026.100408 (PMC13571382; doi:10.1016/j.esmogo.2026.100408)
Supplement: Supplementary file 6 [file mmc6.docx]

| **Characteristic** | **1a (0% residual tumor)**, N = 24^1^ | **1b (<10% residual tumor)**, N = 20^1^ | **2 (10–50% residual tumor)**, N = 26^1^ | **3 (>50% residual tumor)**, N = 53^1^ | **p-value**^2^ |
| --- | --- | --- | --- | --- | --- |
| **Sex** |  |  |  |  | 0.3 |
| Female | 4 (17%) | 2 (10%) | 8 (31%) | 9 (17%) |  |
| Male | 20 (83%) | 18 (90%) | 18 (69%) | 44 (83%) |  |
| **Age category** |  |  |  |  | 0.032 |
| <=45 | 1 (4%) | 2 (10%) | 0 (0%) | 4 (8%) |  |
| >=65 | 18 (75%) | 9 (45%) | 9 (35%) | 31 (58%) |  |
| >45 <65 | 5 (21%) | 9 (45%) | 17 (65%) | 18 (34%) |  |
| **Year of diagnosis** |  |  |  |  | 0.5 |
| 2005-2009 | 1 (4%) | 1 (5%) | 1 (4%) | 2 (4%) |  |
| 2010-2014 | 10 (42%) | 6 (30%) | 5 (19%) | 16 (30%) |  |
| 2015-2019 | 7 (29%) | 7 (35%) | 10 (38%) | 27 (51%) |  |
| 2020-2024 | 6 (25%) | 6 (30%) | 10 (38%) | 8 (15%) |  |
| **BMI** |  |  |  |  | 0.6 |
| normal weight (18.5–24.9) | 6 (30%) | 9 (53%) | 11 (48%) | 16 (42%) |  |
| obesity (≥30.0) | 2 (10%) | 1 (6%) | 5 (22%) | 5 (13%) |  |
| overweight (25.0–29.9) | 12 (60%) | 7 (41%) | 6 (26%) | 16 (42%) |  |
| underweight (<18.5) | 0 (0%) | 0 (0%) | 1 (4%) | 1 (3%) |  |
| (Missing) | 4 | 3 | 3 | 15 |  |
| **Alcohol consumption** |  |  |  |  | 0.4 |
| Alcohol abuse | 4 (17%) | 1 (6%) | 3 (15%) | 2 (4%) |  |
| Moderate alcohol consumption | 10 (43%) | 6 (33%) | 10 (50%) | 19 (42%) |  |
| No alcohol consumption | 9 (39%) | 11 (61%) | 7 (35%) | 24 (53%) |  |
| (Missing) | 1 | 2 | 6 | 8 |  |
| **Nicotine abuse** |  |  |  |  | 0.3 |
| Abuse | 12 (52%) | 12 (63%) | 16 (80%) | 26 (59%) |  |
| No | 11 (48%) | 7 (37%) | 4 (20%) | 18 (41%) |  |
| (Missing) | 1 | 1 | 6 | 9 |  |
| **Vital status** |  |  |  |  | 0.2 |
| Alive | 15 (63%) | 12 (60%) | 15 (58%) | 21 (40%) |  |
| Deceased | 9 (38%) | 8 (40%) | 11 (42%) | 32 (60%) |  |
| **Tumor location** |  |  |  |  | 0.9 |
| Esophagus | 4 (17%) | 3 (15%) | 5 (19%) | 10 (19%) |  |
| GEJ | 15 (63%) | 13 (65%) | 14 (54%) | 26 (49%) |  |
| Stomach | 5 (21%) | 4 (20%) | 7 (27%) | 17 (32%) |  |
| **Stage** |  |  |  |  | 0.5 |
| 2 | 4 (17%) | 1 (5%) | 3 (12%) | 5 (9%) |  |
| 2-3 | 6 (25%) | 7 (35%) | 11 (42%) | 26 (49%) |  |
| 3 | 14 (58%) | 12 (60%) | 12 (46%) | 22 (42%) |  |
| **Signet ring cells** |  |  |  |  | 0.4 |
| negative | 18 (86%) | 12 (67%) | 18 (72%) | 34 (65%) |  |
| positive | 3 (14%) | 6 (33%) | 7 (28%) | 18 (35%) |  |
| (Missing) | 3 | 2 | 1 | 1 |  |
| **MMR** |  |  |  |  | <0.001 |
| deficient | 4 (50%) | 1 (13%) | 2 (13%) | 0 (0%) |  |
| proficient | 4 (50%) | 7 (88%) | 14 (88%) | 32 (100%) |  |
| (Missing) | 16 | 12 | 10 | 21 |  |
| **PD-L1** |  |  |  |  | 0.051 |
| negative | 0 (0%) | 2 (25%) | 0 (0%) | 7 (41%) |  |
| positive | 4 (100%) | 6 (75%) | 11 (100%) | 10 (59%) |  |
| (Missing) | 20 | 12 | 15 | 36 |  |
| **HER2** |  |  |  |  | 0.10 |
| negative | 13 (93%) | 13 (65%) | 20 (91%) | 32 (76%) |  |
| positive | 1 (7%) | 7 (35%) | 2 (9%) | 10 (24%) |  |
| (Missing) | 10 | 0 | 4 | 11 |  |
| **TRG (Mandard)** |  |  |  |  | <0.001 |
| 1 (no residual cancer cell) | 23 (100%) | 0 (0%) | 0 (0%) | 0 (0%) |  |
| 2 (rare residual cancer cells) | 0 (0%) | 17 (94%) | 5 (20%) | 0 (0%) |  |
| 3 (more residual cancer cells, but outgrown by fibrosis) | 0 (0%) | 1 (6%) | 19 (76%) | 1 (2%) |  |
| 4 (residual cancer cells outgrowing fibrosis) | 0 (0%) | 0 (0%) | 1 (4%) | 32 (63%) |  |
| 5 (absence of regressive changes) | 0 (0%) | 0 (0%) | 0 (0%) | 18 (35%) |  |
| (Missing) | 1 | 2 | 1 | 2 |  |
| **ypT** |  |  |  |  | <0.001 |
| 0 | 21 (95%) | 1 (5%) | 0 (0%) | 0 (0%) |  |
| 1 | 0 (0%) | 1 (5%) | 6 (23%) | 9 (17%) |  |
| 2 | 0 (0%) | 10 (50%) | 6 (23%) | 9 (17%) |  |
| 3 | 1 (5%) | 8 (40%) | 13 (50%) | 31 (58%) |  |
| 4 | 0 (0%) | 0 (0%) | 1 (4%) | 4 (8%) |  |
| (Missing) | 2 | 0 | 0 | 0 |  |
| **ypN** |  |  |  |  | 0.005 |
| 0 | 21 (95%) | 11 (55%) | 18 (69%) | 20 (38%) |  |
| 1 | 1 (5%) | 2 (10%) | 3 (12%) | 12 (23%) |  |
| 2 | 0 (0%) | 3 (15%) | 3 (12%) | 11 (21%) |  |
| 3 | 0 (0%) | 4 (20%) | 2 (8%) | 9 (17%) |  |
| (Missing) | 2 | 0 | 0 | 1 |  |
| **ypM** | 0 (0%) | 0 (0%) | 0 (0%) | 0 (0%) |  |
| (Missing) | 21 | 19 | 25 | 49 |  |
| **ypL** | 0 (0%) | 7 (41%) | 6 (26%) | 27 (56%) | <0.001 |
| (Missing) | 10 | 3 | 3 | 5 |  |
| **ypV** | 0 (0%) | 1 (6%) | 1 (4%) | 11 (21%) | 0.047 |
| (Missing) | 10 | 2 | 3 | 1 |  |
| **ypPn** | 0 (0%) | 3 (20%) | 3 (27%) | 21 (54%) | 0.006 |
| (Missing) | 15 | 5 | 15 | 14 |  |
| **R** | 0 (0%) | 0 (0%) | 2 (8%) | 2 (4%) | 0.4 |
| (Missing) | 4 | 1 | 1 | 1 |  |
| **Recurrence** |  |  |  |  | 0.3 |
| No recurrence | 17 (71%) | 12 (60%) | 15 (58%) | 25 (47%) |  |
| Recurrence | 7 (29%) | 8 (40%) | 11 (42%) | 28 (53%) |  |
| **Treatment strategy** |  |  |  |  | 0.2 |
| neoadjuvant | 19 (79%) | 12 (60%) | 13 (50%) | 35 (66%) |  |
| perioperative | 5 (21%) | 8 (40%) | 13 (50%) | 18 (34%) |  |
| **Neoadjuvant therapy** |  |  |  |  | <0.001 |
| Chemo (FLOT) | 5 (21%) | 6 (30%) | 17 (65%) | 17 (32%) |  |
| Chemo (not FLOT) | 11 (46%) | 5 (25%) | 6 (23%) | 26 (49%) |  |
| Chemo+Anti-HER2 | 0 (0%) | 0 (0%) | 0 (0%) | 3 (6%) |  |
| Chemo+Anti-PD-L1 | 1 (4%) | 1 (5%) | 1 (4%) | 0 (0%) |  |
| Immunotherapy | 3 (13%) | 0 (0%) | 0 (0%) | 0 (0%) |  |
| Radiochemotherapy | 4 (17%) | 8 (40%) | 2 (8%) | 4 (8%) |  |
| unknown | 0 (0%) | 0 (0%) | 0 (0%) | 3 (6%) |  |
| ^1^n (%) | | | | | |
| ^2^Pearson's Chi-squared test | | | | | |
