# Supplementary material for: Tumor regression grades in locally advanced gastroesophageal adenocarcinoma: associations with survival, pathological risk factors, and molecular biomarkers in a 19-year real-world cohort
Source: ESMO Gastrointest Oncol. 2026 Sep 2;13(Pt B):100408. doi: 10.1016/j.esmogo.2026.100408 (PMC13571382; doi:10.1016/j.esmogo.2026.100408)
Supplement: Supplementary file 7 [file mmc7.docx]

| **Characteristic** | **1 (no residual cancer cell)**, N = 28^1^ | **2 (rare residual cancer cells)**, N = 31^1^ | **3 (more residual cancer cells, but outgrown by fibrosis)**, N = 41^1^ | **4 (residual cancer cells outgrowing fibrosis)**, N = 46^1^ | **5 (absence of regressive changes)**, N = 23^1^ | **p-value**^2^ |
| --- | --- | --- | --- | --- | --- | --- |
| **Sex** |  |  |  |  |  | 0.3 |
| Female | 5 (18%) | 3 (10%) | 12 (29%) | 9 (20%) | 3 (13%) |  |
| Male | 23 (82%) | 28 (90%) | 29 (71%) | 37 (80%) | 20 (87%) |  |
| **Age category** |  |  |  |  |  | 0.3 |
| <=45 | 1 (4%) | 2 (6%) | 2 (5%) | 5 (11%) | 1 (4%) |  |
| >=65 | 19 (68%) | 12 (39%) | 17 (41%) | 25 (54%) | 13 (57%) |  |
| >45 <65 | 8 (29%) | 17 (55%) | 22 (54%) | 16 (35%) | 9 (39%) |  |
| **Year of diagnosis** |  |  |  |  |  | 0.10 |
| 2005-2009 | 1 (4%) | 2 (6%) | 0 (0%) | 2 (4%) | 0 (0%) |  |
| 2010-2014 | 9 (32%) | 7 (23%) | 4 (10%) | 13 (28%) | 4 (17%) |  |
| 2015-2019 | 8 (29%) | 9 (29%) | 13 (32%) | 21 (46%) | 9 (39%) |  |
| 2020-2024 | 10 (36%) | 13 (42%) | 24 (59%) | 10 (22%) | 10 (43%) |  |
| **BMI** |  |  |  |  |  | 0.4 |
| normal weight (18.5–24.9) | 5 (21%) | 14 (50%) | 22 (59%) | 17 (49%) | 8 (44%) |  |
| obesity (≥30.0) | 4 (17%) | 3 (11%) | 5 (14%) | 5 (14%) | 3 (17%) |  |
| overweight (25.0–29.9) | 15 (63%) | 11 (39%) | 9 (24%) | 12 (34%) | 7 (39%) |  |
| underweight (<18.5) | 0 (0%) | 0 (0%) | 1 (3%) | 1 (3%) | 0 (0%) |  |
| (Missing) | 4 | 3 | 4 | 11 | 5 |  |
| **Alcohol consumption** |  |  |  |  |  | 0.4 |
| Alcohol abuse | 5 (19%) | 3 (10%) | 7 (20%) | 3 (7%) | 0 (0%) |  |
| Moderate alcohol consumption | 12 (44%) | 12 (41%) | 15 (43%) | 21 (50%) | 6 (35%) |  |
| No alcohol consumption | 10 (37%) | 14 (48%) | 13 (37%) | 18 (43%) | 11 (65%) |  |
| (Missing) | 1 | 2 | 6 | 4 | 6 |  |
| **Nicotine abuse** |  |  |  |  |  | 0.6 |
| Abuse | 14 (52%) | 19 (66%) | 25 (69%) | 25 (61%) | 9 (53%) |  |
| No | 13 (48%) | 10 (34%) | 11 (31%) | 16 (39%) | 8 (47%) |  |
| (Missing) | 1 | 2 | 5 | 5 | 6 |  |
| **Vital status** |  |  |  |  |  | 0.070 |
| Alive | 20 (71%) | 20 (65%) | 27 (66%) | 26 (57%) | 8 (35%) |  |
| Deceased | 8 (29%) | 11 (35%) | 14 (34%) | 20 (43%) | 15 (65%) |  |
| **Tumor location** |  |  |  |  |  | 0.8 |
| Esophagus | 6 (21%) | 5 (16%) | 11 (27%) | 7 (15%) | 6 (26%) |  |
| GEJ | 17 (61%) | 19 (61%) | 19 (46%) | 24 (52%) | 12 (52%) |  |
| Stomach | 5 (18%) | 7 (23%) | 11 (27%) | 15 (33%) | 5 (22%) |  |
| **Stage** |  |  |  |  |  | 0.8 |
| 2 | 5 (18%) | 6 (19%) | 8 (20%) | 5 (11%) | 4 (17%) |  |
| 2-3 | 7 (25%) | 10 (32%) | 12 (29%) | 20 (43%) | 9 (39%) |  |
| 3 | 16 (57%) | 15 (48%) | 21 (51%) | 21 (46%) | 10 (43%) |  |
| **Signet ring cells** |  |  |  |  |  | 0.4 |
| negative | 21 (84%) | 22 (76%) | 26 (74%) | 26 (63%) | 16 (80%) |  |
| positive | 4 (16%) | 7 (24%) | 9 (26%) | 15 (37%) | 4 (20%) |  |
| (Missing) | 3 | 2 | 6 | 5 | 3 |  |
| **MMR** |  |  |  |  |  | 0.063 |
| deficient | 4 (33%) | 1 (6%) | 2 (7%) | 2 (7%) | 1 (6%) |  |
| proficient | 8 (67%) | 15 (94%) | 28 (93%) | 28 (93%) | 16 (94%) |  |
| (Missing) | 16 | 15 | 11 | 16 | 6 |  |
| **PD-L1** |  |  |  |  |  | 0.059 |
| negative | 0 (0%) | 1 (7%) | 0 (0%) | 3 (20%) | 3 (25%) |  |
| positive | 8 (100%) | 13 (93%) | 26 (100%) | 12 (80%) | 9 (75%) |  |
| (Missing) | 20 | 17 | 15 | 31 | 11 |  |
| **HER2** |  |  |  |  |  | 0.3 |
| negative | 18 (95%) | 19 (70%) | 32 (82%) | 30 (79%) | 14 (74%) |  |
| positive | 1 (5%) | 8 (30%) | 7 (18%) | 8 (21%) | 5 (26%) |  |
| (Missing) | 9 | 4 | 2 | 8 | 4 |  |
| **TRG (Becker)** |  |  |  |  |  | <0.001 |
| 1a (0% residual tumor) | 23 (100%) | 0 (0%) | 0 (0%) | 0 (0%) | 0 (0%) |  |
| 1b (<10% residual tumor) | 0 (0%) | 17 (77%) | 1 (5%) | 0 (0%) | 0 (0%) |  |
| 2 (10–50% residual tumor) | 0 (0%) | 5 (23%) | 19 (90%) | 1 (3%) | 0 (0%) |  |
| 3 (>50% residual tumor) | 0 (0%) | 0 (0%) | 1 (5%) | 32 (97%) | 18 (100%) |  |
| (Missing) | 5 | 9 | 20 | 13 | 5 |  |
| **ypT** |  |  |  |  |  | <0.001 |
| 0 | 26 (100%) | 1 (3%) | 0 (0%) | 0 (0%) | 0 (0%) |  |
| 1 | 0 (0%) | 5 (16%) | 7 (17%) | 5 (11%) | 6 (27%) |  |
| 2 | 0 (0%) | 12 (39%) | 11 (27%) | 8 (17%) | 2 (9%) |  |
| 3 | 0 (0%) | 13 (42%) | 22 (54%) | 30 (65%) | 12 (55%) |  |
| 4 | 0 (0%) | 0 (0%) | 1 (2%) | 3 (7%) | 2 (9%) |  |
| (Missing) | 2 | 0 | 0 | 0 | 1 |  |
| **ypN** |  |  |  |  |  | 0.007 |
| 0 | 24 (92%) | 19 (61%) | 24 (60%) | 15 (34%) | 9 (41%) |  |
| 1 | 2 (8%) | 4 (13%) | 6 (15%) | 10 (23%) | 6 (27%) |  |
| 2 | 0 (0%) | 5 (16%) | 6 (15%) | 9 (20%) | 4 (18%) |  |
| 3 | 0 (0%) | 3 (10%) | 4 (10%) | 10 (23%) | 3 (14%) |  |
| (Missing) | 2 | 0 | 1 | 2 | 1 |  |
| **ypM** | 0 (0%) | 0 (0%) | 0 (0%) | 0 (0%) | 0 (0%) |  |
| (Missing) | 25 | 26 | 37 | 42 | 20 |  |
| **ypL** | 0 (0%) | 9 (35%) | 13 (36%) | 27 (64%) | 11 (58%) | <0.001 |
| (Missing) | 11 | 5 | 5 | 4 | 4 |  |
| **ypV** | 0 (0%) | 0 (0%) | 5 (13%) | 9 (20%) | 5 (23%) | 0.027 |
| (Missing) | 11 | 2 | 3 | 1 | 1 |  |
| **ypPn** | 0 (0%) | 3 (14%) | 11 (48%) | 19 (54%) | 9 (64%) | <0.001 |
| (Missing) | 19 | 10 | 18 | 11 | 9 |  |
| **R** | 0 (0%) | 1 (3%) | 3 (9%) | 4 (10%) | 1 (5%) | 0.5 |
| (Missing) | 4 | 1 | 6 | 4 | 3 |  |
| **Recurrence** |  |  |  |  |  | 0.2 |
| No recurrence | 22 (79%) | 20 (65%) | 23 (56%) | 27 (59%) | 11 (48%) |  |
| Recurrence | 6 (21%) | 11 (35%) | 18 (44%) | 19 (41%) | 12 (52%) |  |
| **Treatment strategy** |  |  |  |  |  | 0.073 |
| neoadjuvant | 21 (75%) | 20 (65%) | 19 (46%) | 33 (72%) | 16 (70%) |  |
| perioperative | 7 (25%) | 11 (35%) | 22 (54%) | 13 (28%) | 7 (30%) |  |
| **Neoadjuvant therapy** |  |  |  |  |  | 0.001 |
| Chemo (FLOT) | 9 (32%) | 13 (42%) | 23 (56%) | 14 (30%) | 7 (30%) |  |
| Chemo (not FLOT) | 11 (39%) | 7 (23%) | 6 (15%) | 21 (46%) | 9 (39%) |  |
| Chemo+Anti-HER2 | 0 (0%) | 1 (3%) | 4 (10%) | 3 (7%) | 2 (9%) |  |
| Chemo+Anti-PD-L1 | 1 (4%) | 0 (0%) | 2 (5%) | 1 (2%) | 2 (9%) |  |
| Immunotherapy | 3 (11%) | 0 (0%) | 0 (0%) | 0 (0%) | 0 (0%) |  |
| Radiochemotherapy | 4 (14%) | 10 (32%) | 5 (12%) | 3 (7%) | 3 (13%) |  |
| unknown | 0 (0%) | 0 (0%) | 1 (2%) | 4 (9%) | 0 (0%) |  |
| ^1^n (%) | | | | | | |
| ^2^Pearson's Chi-squared test | | | | | | |
